# The Effects of the COVID-19 Pandemic on Pediatric Orthopedic Injuries: A Single-Center Retrospective Study

**DOI:** 10.3390/children11101265

**Published:** 2024-10-19

**Authors:** Betina Hinckel, Sazid Hasan, Clark Yin, Jimmy Lau, Saeed Saleh, Ehab Saleh

**Affiliations:** 1 William Beaumont School of Medicine, Oakland University, Rochester, MI 48309, USA; betina.hinckel@corewellhealth.org (B.H.); sazid.hasan@utoledo.edu (S.H.); ehab.saleh3@corewellhealth.org (E.S.); 2 Department of Orthopedic Surgery, William Beaumont University Hospital, Royal Oak, MI 48073, USA; clark.yin@corewellhealth.org; 3 College of Natural Science, Michigan State University, East Lansing, MI 48824, USA; salehsae@msu.edu

**Keywords:** COVID-19, orthopedic injury, pediatric trauma score, pediatrics

## Abstract

Objectives: The COVID-19 pandemic has had a profound effect on the pediatric population worldwide. The consequences of the lockdown and the reallocation of hospital resources have caused notable changes in the presentation of pediatric orthopedic injuries. Through our use of patient records, we were able to display the epidemiological trends, as well as analyze the changes in the type and mechanism of various injuries. Our purpose is to further understand the considerable effects of the COVID-19 pandemic on pediatric orthopedic trauma and help guide the allocation of future healthcare resources. Methods: We conducted a retrospective cohort study on pediatric patients admitted for any orthopedic injury over a 3-year period (September 2018 to August 2021) at a Midwest level 1 trauma center. Cases were stratified into two groups based on the United States’ COVID-19 lockdown (19 March 2020): Pre-COVID-19 cases were any cases prior to the lockdown and Intra-COVID-19 which are cases following the lockdown. Numerical data and categorical variables were summarized and differences between the case groups were examined using either two-Proportion Z-Test, Independent Two-Sample *t*-test, Pearson’s chi-squared, or Fisher’s exact tests. Results: A total of 3179 pediatric orthopedic referrals occurred between the Pre-COVID-19 and Intra-COVID-19 study periods. We observed a general decrease in orthopedic injuries following the COVID-19 lockdowns with 1235 injuries compared to 1606 Pre-COVID-19. Patterns in the locations of injuries changed; notably, fractures of the humerus, tibia, and fibula decreased significantly (*p* < 0.05). Several mechanisms of injuries decreased significantly following the onset of COVID-19 including injuries caused by monkey bars, basketball, and automobiles (*p* < 0.01). There was a significant increase in the overall injury severity during the Intra-COVID-19 period (*p* < 0.05). Conclusions: Although there was a reduction in acute orthopedic trauma referrals, many injury mechanisms displayed similar trends regardless of restrictions. We clinically observed an overall increase in the severity of pediatric orthopedic injuries during the COVID-19 pandemic.

## 1. Introduction

The novel coronavirus-2019 (COVID-19) pandemic has caused significant disruption to the fields of orthopedic and trauma surgery worldwide. The incidence of pediatric orthopedic injuries Pre-COVID-19 varied depending on location, with sources citing a range of 12 to 36 fractures per 1000 children per year [1,2,3]. A dramatic reduction in the overall orthopedic operations of 44% was noted in a large multi-center study based out of Hong Kong, with elective procedures reduced by 74% to 84% and emergent fractures reduced by 20% to 23% [4]. Christey et al. noted a similar trend in the decreased overall volume of injury admissions in New Zealand during the lockdown. Ohm et al. found a 43% reduction in injured patients during the first three weeks of lockdown in Norway [5,6]. Markiewitz et al. conducted a database review that revealed a 27% decline in monthly pediatric fractures in the United States during the COVID-19 pandemic [7].

This reduction has also been noted among pediatric orthopedic patients worldwide, mostly in the early phases of the pandemic with more restrictive lockdowns [7,8,9,10,11]. Sheridan et al. looked at the number of admissions and surgical procedures for acute pediatric trauma in a level 1 trauma center in Ireland from 2009 to 2020 and noted the lowest number occurred during the 2020 lockdown [10]. Johnson et al. noted a fourfold reduction in common pediatric musculoskeletal injuries associated with organized sports in the United States [6,12]. However, the literature on pediatric orthopedic patients in the United States is sparse, with even less information on racial and socioeconomic factors [13,14].

Studies analyzing the trends in orthopedic injuries and the subsequent management in light of the COVID-19 pandemic predominantly explore trends in the adult population, whilst the literature regarding pediatric injuries is sparse. While the overall number of pediatric orthopedic trauma patients decreased during the height of the pandemic, a few studies demonstrated a decrease in the average age of children experiencing fractures [13,14,15]. Furthermore, given the hesitancy to seek treatment, Shaw et al. noted severe fractures were more likely in older children, an increased rate of non-accidental trauma, and a larger proportion of patients experiencing at least a 5-day delay to definitive treatment [14]. A hesitancy to seek treatment can allow fractures to worsen over time through improper healing, the misalignment of bones, or possible injury to the surrounding tissue. Older children are also more active and perceive pain differently (possibly more resilient) which exacerbates the severity of their fractures. In addition, from surgical supply chain shortages to novel sterile techniques, orthopedic treatment modalities have also been impacted by the pandemic [16,17]. A systematic review and meta-analysis by Lim et al. found a statistically significant higher 30-day mortality [18].

The aim of this study was to

1. Characterize trends and changes in pediatric orthopedic trauma with regard to
  - Mechanism of injury;
  - Injury severity;
  - Management in the context of the COVID-19 pandemic.
2. Provide clinicians with important contextual insight to aid their management of these patients whilst highlighting the overall implications of the pandemic on the field.

## 2. Methodology

This study was approved by our hospital’s institutional review board. We conducted a retrospective cohort study on pediatric patients aged 1 day to 16 years old, admitted over the past 3 years (September 2018 to August 2021) at a Midwest level 1 trauma center for the treatment of any upper extremity, lower extremity, or spine injury; orthopedic related admission; and orthopedic surgery consult. Cases were stratified into two groups based on the exact day of the United States’ first COVID-19 lockdown (19 March 2020). Data from the cases prior to the lockdown were labeled as Pre-COVID-19 and the cases following the lockdown were considered Intra-COVID-19.

From each patient, data collection included demographic information, diagnosis, injury details (e.g., mechanism, location, vitals, and length of stay), procedures performed, number of procedures, complications, mortality, and medications. Injuries and procedures were represented by the ICD-9 and ICD-10 codes. Two authors independently coded each diagnosis and procedure by anatomic location and type (bony and soft tissue). Fractures and dislocations were defined as bony injuries while vascular, ligamentous, tendinous, muscular, skin, and nerve injuries were defined as soft-tissue injuries. Severity scores were calculated for each patient based on the Pediatric Trauma Score: a higher score indicates a less severe injury, while a lower score indicates greater severity [19].

Numerical data and categorical variables were summarized and the normality of the distribution of data was evaluated using the Anderson–Darling normality test. Differences between the case groups for demographics, injury mechanism, injury anatomic location, setting of injury, injury severity, and treatment were calculated using a two-Proportion Z-Test or an Independent Two-Sample *t*-test. Pearson’s chi-squared or Fisher’s exact tests were used to compare categorical data between groups. *p* < 0.05 denoted statistical significance for all the analyses. The analyses were performed using Microsoft Excel v. 2103 (Microsoft Corporation, Redmond, WA, USA).

## 3. Results

A total of 3179 pediatric patients presented with an orthopedic-related injury during the period between 18 September 2018 and 31 August 2021 (Table 1). The period of 18 September 2018 to 12 March 2020 represents the Pre-COVID-19 aggregate, while the Intra-COVID-19 aggregate is represented by the period of 13 March 2020 to 31 August 2021. There were a total of 1708 patients (53.7%) during the Pre-COVID period and 1471 patients (46.3%) that were referred during the Intra-COVID-19 era. During both the Pre-COVID-19 and Intra-COVID-19 era there was a smaller female population, a total of 365 (11.5%), compared to the male population, a total of 2814 (88.5%). Our study also showed that there was a statistically significant difference in the average male BMI between the two study periods, with the Pre-COVID-19 population average BMI of 20.44 and Intra-COVID-19 average BMI of 21.33 (*p* < 0.0001).

**Figure 1:**
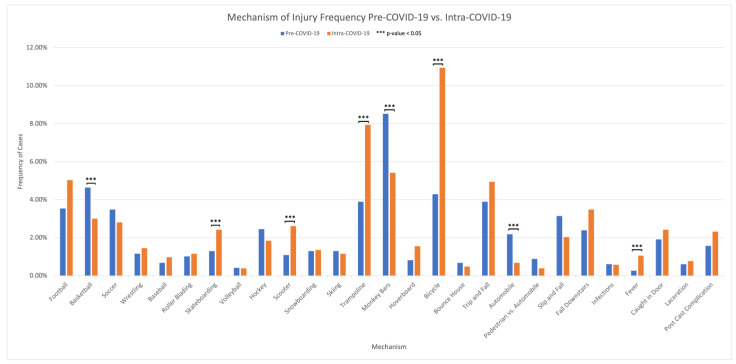
Mechanisms of injury comparison between Pre-COVID-19 (18 September–20 March) and Intra-COVID-19 (20 March–21 August) cohorts.

When comparing the Pre-COVID-19 and Intra-COVID-19 aggregates, we see notable changes in the trends in the mechanism of injury (Figure 1). The most common mechanisms of injury Pre-COVID-19 were due to monkey bars (8.52%), basketball (4.64%), bicycle (4.29%), trampoline (3.89%), or trip and fall (3.89%). This distribution of mechanisms of injury changed in Intra-COVID-19 with bicycle injuries (10.94%) becoming the most common cause of injury, followed by trampoline injuries (7.94%), monkey bars (5.42%), football (5.03%), and trip and fall injuries (4.94%). Statistically significant differences in frequencies were found in the frequency of injuries related to bicycles, basketball, trampolines, and monkey bars. The other mechanisms of injury that experienced statistically significant differences between the two time periods were skateboarding (+1.12%), scooter (1.52%), and motor vehicle collision (−1.50%). The most notable least common mechanisms of injury overall during the Intra-COVID-19 period were pedestrian vs. automobile injuries (*n* = 4, 0.4%), followed by bounce house injuries (*n* = 5, 0.5%), and motor vehicle collision (*n* = 7, 0.7%).

**Figure 2:**
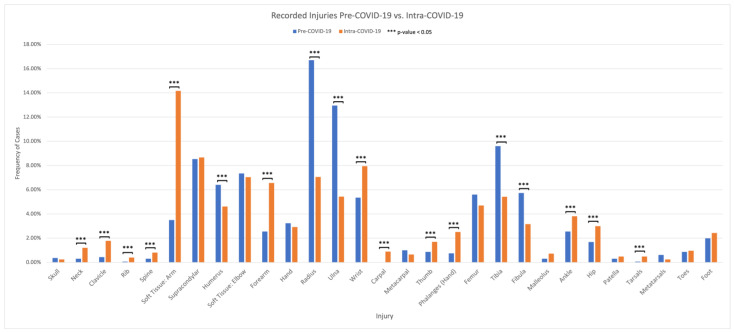
Anatomical location of injury comparison between Pre-COVID-19 and Intra-COVID-19 cohorts.

There were statistically significant increases (*p* < 0.05) in the frequency of the following sites of injury from the Pre-COVID-19 to Intra-COVID-19 study period: soft tissue of the arm (+10.7%), forearm (+4.0%), wrist (+2.6%), hand phalanges (+1.8%), clavicle (+1.4%), hip (+1.3%), ankle (+1.3%), neck (+0.9%), carpal (+0.9%), thumb (+0.8%), spine (+0.5%), tarsals (+0.4%), and rib (+0.3%) (Figure 2). Conversely, there were statistically significant (*p* < 0.05) decreases in the following sites of injury: radius (−9.6%), tibia (−4.1%), fibula (−2.6%), and humerus (−1.8%).

The most common site of injury overall during the Pre-COVID-19 period was radius (*n* = 268, 16.7%), ulna (*n* = 208, 13.0%), tibia (*n* = 154, 9.6%), supracondylar (*n* = 137, 8.5%), and soft tissue of the elbow (*n* = 118, 7.3%) (Figure 2). During the Intra-COVID-19 period, the most common site of injury was soft tissue of the arm (musculocutaneous injuries) (*n* = 175, 14.2%), supracondylar humerus (*n* = 107, 8.7%), wrist (*n* = 98, 7.9%), radius (*n* = 87, 7.0%), and soft tissue of the elbow (*n* = 87, 7.0%). The least common sites of injury overall during the Pre-COVID-19 period were tarsal (*n* = 1, 0.1%), and rib fractures (*n* = 1, 0.1%). During the Intra-COVID-19 period, the least common sites of injury were the metatarsals (*n* = 3, 0.2%), skull (*n* = 3, 0.2%), and rib (*n* = 5, 0.4%).

**Figure 3:**
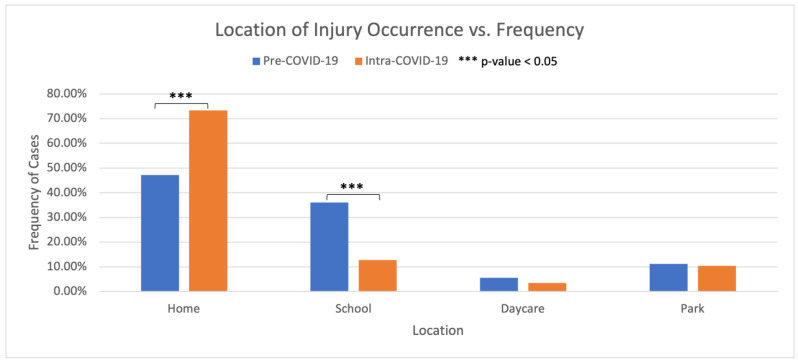
Location of injury comparison between Pre-COVID-19 and Intra-COVID-19 cohorts.

A statistically significant increase of +26.1% in injury occurrence at home during the Intra-COVID-19 period was observed compared to Pre-COVID-19 (*p* < 0.05) (Figure 3). In contrast, a 23.3% decrease (*p* < 0.05) in injury occurrence at school was observed during the same period.

Treatment trends were analyzed and a decrease in the prescription of naproxen (−1.3%), oxycodone (−1.1%), tramadol (−0.7%), and ibuprofen (−0.4%) were observed between the time periods (*p* > 0.05). However, there was an increase in the prescription of aspirin, fentanyl, and methadone. Casting, splinting, and reductions were more common during the Intra-COVID-19 period. The most common treatment modality during the Pre-COVID-19 period was Tylenol (34.5%), followed by splinting (29.6%), and casting (11%). During the Intra-COVID-19 period, the most common treatment modalities were Tylenol and splinting (31.6%), followed by casting (13.0%).

**Figure 4:**
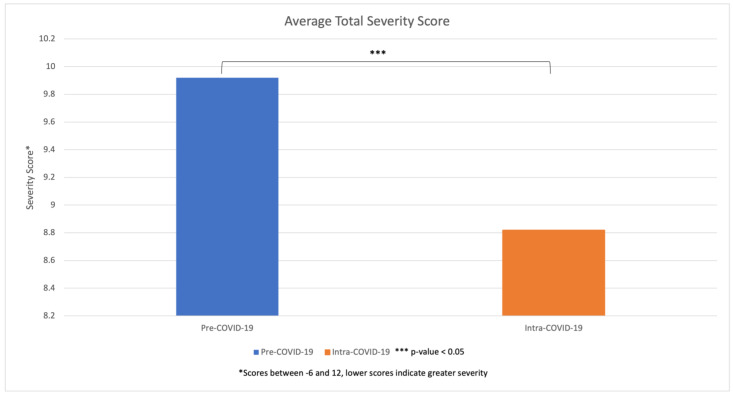
Comparison of average total severity score based on the Pediatric Trauma Score between Pre-COVID-19 and Intra-COVID-19 cohorts.

**Figure 5:**
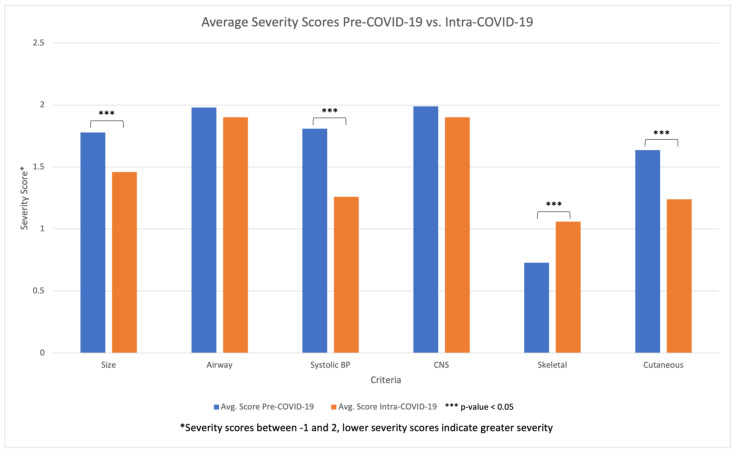
Comparison of average severity score stratified by Pediatric Trauma Score criteria between Pre-COVID-19 and Intra-COVID-19 cohorts.

The Pediatric Trauma Score (PTS) comprises many parameters that assess the pediatric traumatic injury. A higher PTS indicates a lower mortality, while a lower PTS is more fatal. Severity scores were assessed which revealed a statistically significant decrease in the Pediatric Trauma Score during the Intra-COVID-19 period, an average score of 8.82, compared to the Pre-COVID-19 period, an average score of 9.92 (Figure 4). Breaking down the individual parameters that make up the scoring scale, we observed a significant decrease in the average severity scores of size/weight, systolic BP, and cutaneous involvement during the Intra-COVID-19 period compared to the Pre-COVID-19 period (Figure 5). However, after reviewing the patients’ medical charts, we observed an increase in the average severity score in skeletal injuries during the Intra-COVID-19 period. The patients with no fractures were given a score of 2, a closed fracture a score of 1, and open/multiple fractures a score of −1. The average skeletal injury score Pre-COVID-19 was 0.72, which increased to 1.06 during Intra-COVID-19. While there was a decrease in skeletal injury severity, there was an overall increase in injury severity. This is due to the scoring system comprising multiple factors (size/weight, airway, systolic BP, central nervous system, skeletal, and cutaneous). The statistically significant increase in the injury of the soft tissue of the arm, which falls into the cutaneous/open wound involvement of the Pediatric Trauma Score, increased the injury severity.

## 4. Discussion

Pediatric fractures represent a significant aspect of the public healthcare system around the world [20,21]. The COVID-19 pandemic had a significant impact on the healthcare system secondary to policies put in place including quarantine, social distancing, and public facility closures. With the social distancing policies, hospitals around the world observed a decrease in the occurrence of traumatic injuries [22,23]. Our study examined the trends in pediatric orthopedic surgery trauma cases presenting to the emergency center at a large level 1 trauma center in the United States. Our most important finding was that despite the overall decrease in pediatric orthopedic trauma after quarantine, we clinically observed an overall concurrent increase in injury severity during the pandemic. This was supported by our data analysis using the Pediatric Trauma Score.

Demographically, there was no statistically significant difference in the male to female ratio of patients presenting with pediatric orthopedic trauma between the two periods, with more males than females. Prior studies noted a dramatic reduction in the overall orthopedic surgeries that occurred during COVID-19, as well as the frequency of overall pediatric orthopedic injuries, which was collaborated by our analysis [3,6,11,24,25,26]. A decrease in the total number of injuries would be expected given the widespread closures of public places [10,13]. Similar to other retrospective studies, our analysis did not show a statistically significant difference in the number of patients presenting between the cohorts [27]. It has been suggested that the fear of the pandemic may also have contributed to the decrease in number and the delay in time to presentation [13,23]. While demonstrated in other specialties, this has not been shown in cases involving pediatric musculoskeletal injuries, with Bram et al. and Johnson et al. finding no significant delay in the presentation of pediatric sports injuries [12,13]. This is in contrast to Shaw et al. who noted a significant increase in the proportion of patients who underwent definitive treatment greater than 5 days after their initial injury [14]. Our study was not designed to look at this factor.

We found that the most common pre-pandemic injury mechanism was monkey bars, while bicycling was the most common injury mechanism Intra-COVID-19. The largest increases in the frequency of the mechanism of injury were trampolining and bicycling, reflecting an increase in injuries resulting from home activities also observed in prior studies [14]. Interestingly, trampoline injuries have recently drawn attention for their danger; Ibrahim et al. found a 400% increase in trampolining injuries during the COVID-19 pandemic compared to 2019, which were responsible for 28% of the surgical cases in a district general hospital in the UK. The authors caution that trampoline injuries should be considered a high-energy mechanism of injury [28]. In general, we found a notable decrease in contact sports-related injuries, similar to previous retrospective studies [27,29,30]. That finding was expected as a result of school and competitive league closures.

There were numerous statistically significant changes in the incidence of specific sites of injuries when comparing the two time periods. Upper extremity fractures were the most common in the Pre-COVID-19 period as well as in the lockdown period, but we noted a decrease in the number of radius fractures. Ruzzini et al. observed a similar trend when looking at pediatric trauma during the lockdown in Italy, reporting 77.6% of the fractures at a pediatric emergency department involving the upper limbs [31]. Shaw et al. also noted upper extremity fractures to be more common when looking at pediatric trauma cases in a level 1 trauma center in Colorado pre-pandemic [14].

The significant increase in the frequency of orthopedic trauma at home can be attributed to social distancing guidelines, which skewed the distribution of the time children spent at home compared to in other settings. This shift in behavior has been echoed in the prior literature including Raitio et al. and Malige et al. which commented on the decrease in school, organized sports-related injuries, and daycare injuries [9,32]. Malige et al. also noted that the increase in home trauma can be attributed to child abuse due to the toll of COVID-19-related factors such as unemployment, social distancing, and increased at-home time [32].

When looking at the severity scoring of injuries, we noted a statistically significant increase in the severity of traumatic injuries during the Intra-COVID-19 period. In contrast, Sanford et al. noted no change in the severity of pediatric trauma cases, while Shaw et al. found greater fracture numbers in younger children and a relative increase in severe fractures in older children [14,29]. This increase in more severe injuries is likely multifactorial, possibly due to the increase in high-energy mechanisms such as trampoline use or the potential hesitancy of parents to bring patients to the hospital for falsely perceived less severe injuries as demonstrated by Shaw et al. [14]. We found an increase in injuries related to sensitive areas such as the neck, spine, and ribs, which could reflect non-accidental trauma (NAT). NAT of the spine and ribs are more likely to be fractured from physical abuse such as shaking and excessive external force. Shaw et al. noted a nonsignificant increase in the odds of a pediatric fracture occurring secondary to NAT, as well as Kovler et al. [14,33]. The increased severity of injuries in our study may explain the changes in the frequency of treatment modalities with a statistically significant decrease in noninvasive medical management such as the use of naproxen, ibuprofen, oxycodone, and tramadol. However, there was no statistically significant difference in procedures such as casting, splinting, and reductions. Hakkenbrak et al. observed higher severity cases and a relative increase in patients who suffered a high-energy trauma and were treated surgically following the onset of the pandemic [34].

Important limitations of our study include the retrospective nature, reliance on the accuracy of the International Classification of Diseases (ICD) diagnosis codes, and only having data from a single center, in addition to the geographic and subsequent demographic limitations that accompany single-center studies. Given the nature of retrospective studies, selection bias is a potential limitation. Incomplete information in patient encounter notes utilized for severity scoring also limited our breadth of analysis, barring us from calculating severity scores for our entire sample size. In future studies, we wish to assess for the suspicion of non-accidental trauma in this population as well as observe trends in child abuse cases. Further multi-center studies are needed with prospective data collection.

## 5. Conclusions

The COVID-19 pandemic and its related countermeasures have had significant impacts on pediatric orthopedic trauma trends with regard to the mechanism, anatomical location, and social setting of injury. While there was a decrease in acute orthopedic trauma referrals during the pandemic lockdown, many injury mechanisms displayed similar trends. We clinically observed an overall increase in the severity of pediatric orthopedic injuries during the COVID-19 pandemic. Further studies should include institutions at different geographic locations, especially in underdeveloped and developing countries, to improve the generalization of the results. In addition, further epidemiological studies are needed to assess the impacts of delayed and virtual care on patient outcomes.

## Figures and Tables

**Table 1:** Pediatric patients presenting with an orthopedic-related injury.

	Pre-COVID-19 18 September 2018–18 March 2020 (*n* = 1708)	Intra-COVID-19 13 March 2020–31 August 2021 (*n* = 1471)	*(p*-Value)
Female, *n* (%)	210 (12.3)	155 (10.5)	>0.05 ^+^
Male, *n* (%)	1499 (87.7)	1316 (89.5)	>0.05 ^+^
Total Average BMI			
Average Male BMI	20.44	21.33	0.0001 *
Average Female BMI	21.09	23.19	>0.05 *
Mean Age, years (SD)	8.36 (4.55)	8.1 (4.64)	>0.05 ^+^
Mean Length of Stay, Days (SD)	0.86 (1.44)	1.34 (19.54)	>0.05 ^+^
Range of Length of Stay (Total Days)	0–15	0–21	
Orientation of Injury			
Left Sided Injury	872	698	0.021 *
Right Sided Injury	699	590	>0.05 *
Bilateral Injury	36	36	>0.05 *

* Two-Proportion Z-Test. ^+^ Unpaired Two-Sample *t*-test. SD: Standard Deviation.

## Data Availability

The data presented in this study are available upon request from the corresponding author. The data are not publicly available due to privacy and ethical concerns.
